# Supplementary material for: Deep-sequencing of viral genomes from a large and diverse cohort of treatment-naive HIV-infected persons shows associations between intrahost genetic diversity and viral load
Source: PLoS Comput Biol. 2023 Jan 3;19(1):e1010756. doi: 10.1371/journal.pcbi.1010756 (PMC9838853; doi:10.1371/journal.pcbi.1010756)
Supplement: S1 Table — IQR (interquartile range). (DOCX) [file pcbi.1010756.s001.docx]

**S1Table.** Characteristics of 2,650 participants from the START trial included in this analysis. IQR (interquartile range).

| **Characteristic** | **START participants after quality control, No. (%) (N=2,650)** |
| --- | --- |
| Age median (IQR), years | 36 (29–44) |
| Sex |  |
| Female | 545 (21%) |
| Male | 2105 (79%) |
| Race/ethnic group |  |
| Asian | 246 (9%) |
| Black | 620 (23%) |
| Hispanic | 357 (14%) |
| White | 1,349 (51%) |
| Other | 78 (3%) |
| Geographic region |  |
| Asia | 226 (9%) |
| Africa | 411 (15%) |
| Australia | 67 (3%) |
| Europe and Israel | 1040 (39%) |
| Latin America | 638 (24%) |
| United States | 268 (10%) |
| Mode of HIV infection |  |
| Injection drug use | 34 (1%) |
| Sex with same sex | 1654 (62%) |
| Sex with opposite sex | 840 (32%) |
| Other | 122 (5%) |
| Subtype |  |
| A | 101 (4%) |
| B | 1543 (58%) |
| C | 198 (8%) |
| AB | 84 (3%) |
| AE | 142 (5%) |
| BC | 79 (3%) |
| Mixed | 76 (3%) |
| Other | 344 (13%) |
| Unable | 83 (3%) |
| Duration of infection |  |
| <6 months | 256 (10%) |
| 6–24 months | 1424 (54%) |
| >24 months | 970 (36%) |
| HIV viral load, median (IQR), copies/mL | 24,276 (8,651–61,964) |
| CD4+ cell count, median (IQR), cells/μL | 638.2 (578.5–739.9) |
