# Supplementary material for: Deep-sequencing of viral genomes from a large and diverse cohort of treatment-naive HIV-infected persons shows associations between intrahost genetic diversity and viral load
Source: PLoS Comput Biol. 2023 Jan 3;19(1):e1010756. doi: 10.1371/journal.pcbi.1010756 (PMC9838853; doi:10.1371/journal.pcbi.1010756)
Supplement: S2 Table — (DOCX) [file pcbi.1010756.s002.docx]

**S2 Table.** HIV genomic positions for which ≤800 samples had a minimum of 500-fold genome sequencing coverage depth at position.

| **Gene** | **AA positions** | **Distance to amplicon end (nt)** |
| --- | --- | --- |
| Pol | 938 | 162–164 |
| Env | 9–14 | 284–297 |
| Env | 137–151 | 666–707 |
| Env | 311–320 | 1188–1214 |
| Env | 349–360 | 1302–1334 |
| Env | 396–410 | 1443–1484 |
| Vpu | 64–68 | 284–297 |
| Rev | 7–9 | 1–9 |
| Tat | 53–55 | 4–12 |
| Nef | 175–180 | 185–199 |
| Nef | 182–207 | 103–177 |
