# Supplementary material for: Deep-sequencing of viral genomes from a large and diverse cohort of treatment-naive HIV-infected persons shows associations between intrahost genetic diversity and viral load
Source: PLoS Comput Biol. 2023 Jan 3;19(1):e1010756. doi: 10.1371/journal.pcbi.1010756 (PMC9838853; doi:10.1371/journal.pcbi.1010756)
Supplement: S3 Table — (DOCX) [file pcbi.1010756.s003.docx]

**S3 Table.** HIV genome’s mean Shannon entropy at the most variable positions.

| **Gene** | **Position** | **Entropy** | **Feature^1^** |
| --- | --- | --- | --- |
| Gag | 373 | 0.3294 | p2 |
| Gag | 374 | 0.3780 | p2 |
| Gag | 375 | 0.3749 | p2 |
| Gag | 376 | 0.3444 | p2 |
| Gag | 483 | 0.3108 | − |
| Pol | 051 | 0.3797 | − |
| Pol | 082 | 0.3202 | − |
| Env | 087 | 0.3363 | − |
| Env | 135 | 0.3381 | V1 |
| Env | 136 | 0.3432 | V1; glycosylation NDT |
| Env | 184 | 0.3067 | V2 |
| Env | 185 | 0.5717 | V2 |
| Env | 186 | 0.6739 | V2; glycosylation NDT |
| Env | 187 | 0.6497 | V2; glycosylation NDT |
| Env | 188 | 0.6614 | V2; glycosylation NDT |
| Env | 189 | 0.5608 | V2 |
| Env | 190 | 0.3961 | V2 |
| Env | 321 | 0.4310 | V3 |
| Env | 335 | 0.3613 | − |
| Env | 336 | 0.3347 | − |
| Env | 337 | 0.3096 | − |
| Env | 340 | 0.3552 | glycosylation NNT |
| Env | 343 | 0.4058 | − |
| Env | 347 | 0.4102 | − |
| Env | 362 | 0.3178 | − |
| Env | 389 | 0.3485 | V4; glycosylation NST |
| Env | 412 | 0.4434 | V4 |
| Env | 460 | 0.7462 | V5 |
| Env | 461 | 0.8018 | V5 |
| Env | 462 | 0.7776 | V5 |
| Env | 463 | 0.6942 | V5; glycosylation NES |
| Env | 464 | 0.7749 | V5; glycosylation NES |
| Env | 465 | 0.4497 | V5; glycosylation NES |
| Env | 620 | 0.3371 | − |
| Env | 624 | 0.3537 | glycosylation NHT |
| Env | 640 | 0.3186 | glycosylation NYT |

^1^ HIV Sequence Compendium 2018: <https://www.hiv.lanl.gov/content/sequence/HIV/COMPENDIUM/2018/hiv1prot.pdf> (accessed: 2022-02-09)
