# Supplementary material for: Deep-sequencing of viral genomes from a large and diverse cohort of treatment-naive HIV-infected persons shows associations between intrahost genetic diversity and viral load
Source: PLoS Comput Biol. 2023 Jan 3;19(1):e1010756. doi: 10.1371/journal.pcbi.1010756 (PMC9838853; doi:10.1371/journal.pcbi.1010756)
Supplement: S4 Table — (DOCX) [file pcbi.1010756.s004.docx]

**S4 Table.** Summary of the total considered positions, explained variance and Pearson’s correlation coefficient based on the infection duration in the robustness analysis of the results when changing the sequencing depth requirement for positions to be included from 500-fold to 300-fold and 1,000-fold, respectively.

|  | | **300x** | **500x** | **1000x** |
| --- | --- | --- | --- | --- |
| **Total positions considered** | | 2,542 | 2,447 | 2,085 |
| **Total samples included** | | 2,783 | 2,650 | 2,406 |
| **High entropy positions (≥0.3)** | | 58 (Gag 373, Gag 374, Gag 375, Gag 376, Gag 483, Pol 051, Pol 082, Pol 980, Env 009, Env 010, Env 011, Env 013, Env 087, Env 135, Env 136, Env 137, Env 138, Env 151, Env 184, Env 185, Env 186, Env 187, Env 188, Env 189, Env 190, Env 311, Env 321, Env 335, Env 336, Env 337, Env 340, Env 343, Env 347, Env 350, Env 353, Env 354, Env 355, Env 356, Env 357, Env 358, Env 360, Env 362, Env 389, Env 396, Env 397, Env 410, Env 412, Env 460, Env 461, Env 462, Env 463, Env 464, Env 465, Env 620, Env 624, Env 640, Vpu 065, Vpu 068) | 36 (Gag 373, Gag 374, Gag 375, Gag 376, Gag 483, Pol 051, Pol 082, Env 087, Env 135, Env 136, Env 184, Env 185, Env 186, Env 187, Env 188, Env 189, Env 190, Env 321, Env 335, Env 336, Env 337, Env 340, Env 343, Env 347, Env 362, Env 389, Env 412, Env 460, Env 461, Env 462, Env 463, Env 464, Env 465, Env 620, Env 624, Env 640) | 22 (Gag 373, Gag 374, Gag 375, Gag 376, Pol 051, Pol 082, Env 087, Env 184, Env 185, Env 186, Env 187, Env 188, Env 189, Env 190, Env 308, Env 335, Env 336, Env 337, Env 340, Env 389, Env 620, Env 624) |
| **Explained variance** | |  |  |  |
|  | **Early infection** | 1.18% | 3.91% | 4.97% |
|  | **Intermediate infection** | 2.36% | 3.56% | 4.84% |
|  | **Late infection** | 9.36% | 10.70% | 11.91% |
| **Pearson’s correlation coefficient** | |  |  |  |
|  | **Early infection** | 0.11 | 0.20 | 0.22 |
|  | **Intermediate infection** | 0.15 | 0.19 | 0.22 |
|  | **Late infection** | 0.31 | 0.33 | 0.35 |
| **VL~Mean sample’s Shannon entropy** | | 3.08% | 4.1% | 4.91% |
| **VL~Mean sample’s Shannon entropy + age + sex + race + duration of infection + PC1 + PC2 + PC3 + PC4** | | 9.54% | 10.3% | 11.24% |
