# Supplementary material for: Deep-sequencing of viral genomes from a large and diverse cohort of treatment-naive HIV-infected persons shows associations between intrahost genetic diversity and viral load
Source: PLoS Comput Biol. 2023 Jan 3;19(1):e1010756. doi: 10.1371/journal.pcbi.1010756 (PMC9838853; doi:10.1371/journal.pcbi.1010756)
Supplement: S5 Table — (DOCX) [file pcbi.1010756.s005.docx]

**S5 Table.** Linear regression analysis of predictors of the viral load (VL).

|  | | **Model 1** | **Model 2** | **Model 3** | **Model 4** | **Model 5** | **Model 6** | **Model 7** | **Model 8** | **Model 9** |
| --- | --- | --- | --- | --- | --- | --- | --- | --- | --- | --- |
| **Intersect** | | 4.11*** | 4.10*** | 4.11*** | 4.19*** | 4.25*** | 4.17*** | 4.14*** | 4.23*** | 4.13*** |
| **Sample’s mean Shannon entropy** | | 4.82*** | 4.82*** | 5.00*** | 4.96*** | 5.86*** | 6.63*** | 6.63*** |  | 6.58*** |
| **Age** | |  | 1.9x10^-4^ | 4.8x10^-4^ | 8.1x10^-4^ | 2.6x10^-3^* | 2.6x10^-3^* | 2.6x10^-3^* |  | 2.7x10^-3^* |
| **Female sex** | |  |  | -0.17*** | -0.13*** | -0.11*** | -0.11*** | -0.11*** |  | -0.11*** |
| **Race** | |  |  |  |  |  |  |  |  |  |
|  | Black |  |  |  | -0.17*** | -0.14** | -0.11 | -0.11 |  | -0.11 |
|  | Hispanic |  |  |  | -0.06 | -0.06 | -3.3x10^-3^ | -2.6x10^-3^ |  | -2.0x10^-3^ |
|  | Other |  |  |  | -0.24** | -0.25*** | -0.21* | -0.21* |  | -0.21* |
|  | White |  |  |  | -0.08 | -0.08 | -0.02 | -0.03 |  | -0.02 |
| **Duration of infection** | |  |  |  |  |  |  |  |  |  |
|  | >24 months |  |  |  |  | -0.32*** | -0.33*** | -0.33*** |  | -0.33*** |
|  | 6–24 months |  |  |  |  | -0.14*** | -0.14*** | -0.14*** |  | -0.15*** |
| **PC1–4** | |  |  |  |  |  |  |  |  |  |
|  | PC1 |  |  |  |  |  | -2.09** | -2.30 |  | -2.0** |
|  | PC2 |  |  |  |  |  | 0.90 | 3.51* |  | 0.90* |
|  | PC3 |  |  |  |  |  | 1.03 | 1.88 |  | 0.95 |
|  | PC4 |  |  |  |  |  | -4.58*** | -4.09*** |  | -4.71*** |
| **Subtype** | |  |  |  |  |  |  |  |  |  |
|  | AB |  |  |  |  |  |  | -0.16 |  |  |
|  | AE |  |  |  |  |  |  | -0.05 |  |  |
|  | B |  |  |  |  |  |  | 0.04 |  |  |
|  | BC |  |  |  |  |  |  | 0.05 |  |  |
|  | C |  |  |  |  |  |  | 0.20 |  |  |
|  | Mixed |  |  |  |  |  |  | 3.2x10^-3^ |  |  |
|  | Other |  |  |  |  |  |  | -0.03 |  |  |
|  | Unable |  |  |  |  |  |  | 0.03 |  |  |
| **Average sequencing depth (positions >500x)** | |  |  |  |  |  |  |  | 3.2x10^-8^*** | 3.2x10^-6^** |
| **F-value** | | - | 0.03 | 35.80 | 5.44 | 37.31 | 12.58 | 1.82 | - | - |
| **Pr(>F)** | | - | 8.7x10^-1^ | 2.5x10^-9^ | 3.6x10^-4^ | <2.2x10^-16^ | 3.8x10^-10^ | 8.0x10^-2^ | - | - |
| **Explained variance** | | 4.1% | 4.1% | 5.4% | 6.1% | 8.6% | 10.3% | 11.0% | 0.68% | 10.7% |

* p<0.05; ** p<0.01; *** p<0.001
