# Supplementary material for: Deep-sequencing of viral genomes from a large and diverse cohort of treatment-naive HIV-infected persons shows associations between intrahost genetic diversity and viral load
Source: PLoS Comput Biol. 2023 Jan 3;19(1):e1010756. doi: 10.1371/journal.pcbi.1010756 (PMC9838853; doi:10.1371/journal.pcbi.1010756)
Supplement: S6 Table — P-values below the 0.05 threshold are marked in bold. (DOCX) [file pcbi.1010756.s006.docx]

**S6 Table.** Joint multiple linear regression model with the Shannon entropy values from significantly associated AA positions. P-values below the 0.05 threshold are marked in bold.

|  | | **Effect size (95% CI)** | **p-value** | **Explained variance (%)** |
| --- | --- | --- | --- | --- |
| **Intercept** | | 4.32 (3.94–4.69) | **<2.2×10^-16^** | — |
| **Pol 34** | | 0.20 | **4.2×10^-2^** | 0.70 |
| **Pol 75** | | 0.07 | 6.1×10^-1^ | 0.38 |
| **Pol 97** | | 0.17 | 5.6×10^-2^ | 1.50 |
| **Pol 195** | | 0.20 | **1.5×10^-2^** | 1.16 |
| **Pol 329** | | 1.3×10^-3^ | 9.9×10^-1^ | 2.6×10^-3^ |
| **Pol 441** | | 0.25 | **2.5×10^-2^** | 1.58 |
| **Env 32** | | 0.13 | **2.1×10^-2^** | 1.33 |
| **Env 87** | | -0.01 | 8.5×10^-1^ | 7.02×10^-5^ |
| **Env 102** | | 0.03 | 8.1×10^-1^ | 0.09 |
| **Env 240** | | -0.11 | 8.5×10^-2^ | 0.24 |
| **Env 336** | | 0.12 | **2.1×10^-2^** | 0.56 |
| **Env 453** | | 0.04 | 7.0×10^-1^ | 0.11 |
| **Env 674** | | 0.16 | **1.8×10^-2^** | 1.22 |
| **Env 775** | | 0.48 | **1.4×10^-4^** | 2.26 |
| **Env 812** | | 0.14 | 1.1×10^-1^ | 0.24 |
| **Nef 15** | | 0.17 | **6.9×10^-3^** | 1.23 |
| **Age** | | 6.5×10^-3^ | **5.5×10^-3^** | 0.93 |
| **Female sex** | | -0.03 | 6.7×10^-1^ | 0.65 |
| **Race** | |  |  | 4.13 |
|  | Black | -0.31 | 8.6×10^-2^ |  |
|  | Hispanic | -0.24 | 1.9×10^-1^ |  |
|  | Other | -0.68 | **2.8×10^-3^** |  |
|  | White | -0.20 | 2.6×10^-1^ |  |
| **Duration of infection** | |  |  | 2.18 |
|  | >24 months | -0.24 | **1.3×10^-2^** |  |
|  | 6–24 months | -0.06 | 4.9×10^-1^ |  |
| **PC1–4** | |  |  |  |
|  | PC1 | -0.81 | 6.5×10^-1^ | 1.46×10^-4^ |
|  | PC2 | -0.04 | 9.8×10^-1^ | 0.04 |
|  | PC3 | 0.52 | 7.8×10^-1^ | 0.04 |
|  | PC4 | -8.24 | **4.9×10^-3^** | 1.33 |
