# Supplementary material for: Deep-sequencing of viral genomes from a large and diverse cohort of treatment-naive HIV-infected persons shows associations between intrahost genetic diversity and viral load
Source: PLoS Comput Biol. 2023 Jan 3;19(1):e1010756. doi: 10.1371/journal.pcbi.1010756 (PMC9838853; doi:10.1371/journal.pcbi.1010756)
Supplement: S7 Table — (DOCX) [file pcbi.1010756.s007.docx]

**S7 Table.** Average RMSE values for the train and test datasets from the 5-fold cross validation analysis for the 16 Shannon entropy HIV AA positions associated with VL.

| **AA** | **RMSE train** | **RMSE test** |
| --- | --- | --- |
| **Pol 34** | 0.59 | 0.58 |
| **Pol 75** | 0.59 | 0.59 |
| **Pol 97** | 0.59 | 0.59 |
| **Pol 195** | 0.59 | 0.58 |
| **Pol 329** | 0.59 | 0.60 |
| **Pol 441** | 0.59 | 0.58 |
| **Env 32** | 0.56 | 0.62 |
| **Env 87** | 0.57 | 0.60 |
| **Env 102** | 0.59 | 0.54 |
| **Env 240** | 0.58 | 0.58 |
| **Env 336** | 0.57 | 0.58 |
| **Env 453** | 0.57 | 0.57 |
| **Env 674** | 0.57 | 0.57 |
| **Env 775** | 0.58 | 0.58 |
| **Env 812** | 0.57 | 0.65 |
| **Nef 15** | 0.58 | 0.55 |
