# Supplementary material for: Deep-sequencing of viral genomes from a large and diverse cohort of treatment-naive HIV-infected persons shows associations between intrahost genetic diversity and viral load
Source: PLoS Comput Biol. 2023 Jan 3;19(1):e1010756. doi: 10.1371/journal.pcbi.1010756 (PMC9838853; doi:10.1371/journal.pcbi.1010756)
Supplement: S8 Table — (DOCX) [file pcbi.1010756.s008.docx]

**S8 Table.** Joint model of multiple linear regression with the consensus AA variant calls which were significantly associated with viral load. P-values below the 0.05 threshold are marked in bold.

|  | | **Effect size** | **p-value** | **Explained variance (%)** |
| --- | --- | --- | --- | --- |
| **Intercept** | | 3.33 | **3.3×10^-4^** | — |
| **Pol 640 A** | | -0.01 | 0.94 | 0.15 |
| **Pol 642 Q** | | 0.13 | 0.31 | 0.03 |
| **Pol 643 D** | | -0.33 | **0.04** | 0.26 |
| **Pol 679 Q** | | -0.10 | **0.03** | 0.65 |
| **Pol 978 R** | | 0.31 | 0.39 | 0.32 |
| **Pol 979 K** | | 0.54 | 0.38 | 0.22 |
| **Pol 980 E** | | 0.75 | 0.35 | 0.01 |
| **Pol 981 K** | | 0.32 | 0.44 | 0.09 |
| **Pol 982 I** | | 0.25 | 0.47 | 0.07 |
| **Rev 6 G** | | 0.16 | 0.15 | 0.12 |
| **Rev 9 D** | | 0.14 | 0.21 | 0.07 |
| **Tat 55 R** | | -0.24 | 0.43 | 0.01 |
| **Tat 85 P** | | 0.09 | 0.20 | 0.02 |
| **Age** | | 4.5×10^-3^ | **3.1×10^-3^** | 0.16 |
| **Female sex** | | -0.13 | **6.2×10^-3^** | 0.75 |
| **Race** | |  |  | 1.11 |
|  | Black | -0.26 | **3.6×10^-3^** |  |
|  | Hispanic | -0.16 | 0.09 |  |
|  | Other | -0.45 | **1.1×10^-4^** |  |
|  | White | -0.17 | **0.05** |  |
| **Duration of infection** | |  |  | 1.56 |
|  | >24 months | -0.21 | **1.1×10^-3^** |  |
|  | 6–24 months | -0.06 | 0.22 |  |
| **PC1–4** | |  |  |  |
|  | PC1 | -1.89 | 0.10 | 0.20 |
|  | PC2 | -2.58 | **0.03** | 0.25 |
|  | PC3 | 1.12 | 0.28 | 0.05 |
|  | PC4 | -1.47 | 0.16 | 0.11 |
