# Supplementary material for: Deep-sequencing of viral genomes from a large and diverse cohort of treatment-naive HIV-infected persons shows associations between intrahost genetic diversity and viral load
Source: PLoS Comput Biol. 2023 Jan 3;19(1):e1010756. doi: 10.1371/journal.pcbi.1010756 (PMC9838853; doi:10.1371/journal.pcbi.1010756)
Supplement: S2 Fig — (DOCX) [file pcbi.1010756.s010.docx]

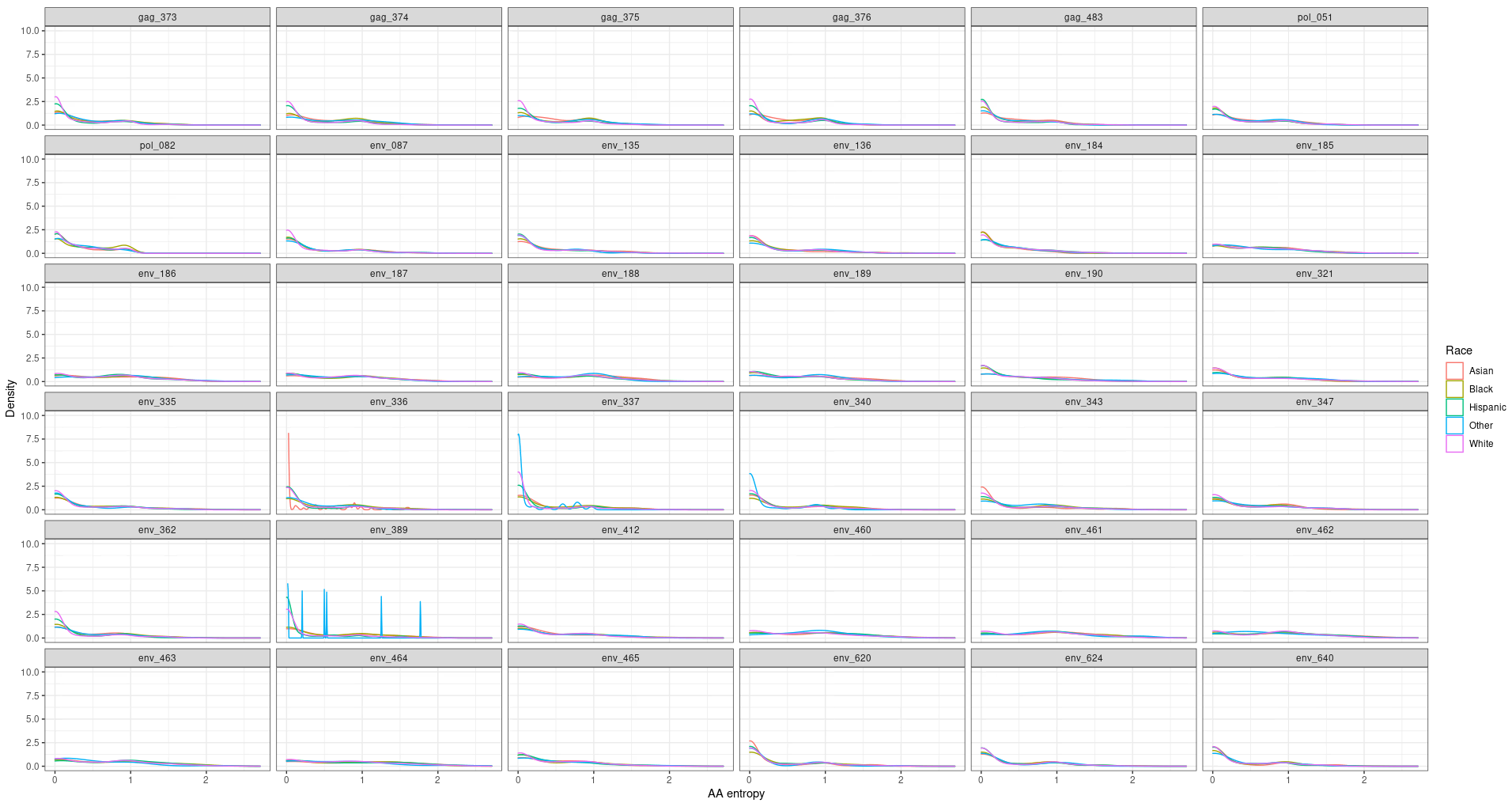


**S2 Fig.** The density plots of the mean Shannon entropy in different races of the 36 most variable positions.
