# Supplementary figures and images for: Deep-sequencing of viral genomes from a large and diverse cohort of treatment-naive HIV-infected persons shows associations between intrahost genetic diversity and viral load
Source: PLoS Comput Biol. 2023 Jan 3;19(1):e1010756. doi: 10.1371/journal.pcbi.1010756 (PMC9838853; doi:10.1371/journal.pcbi.1010756)

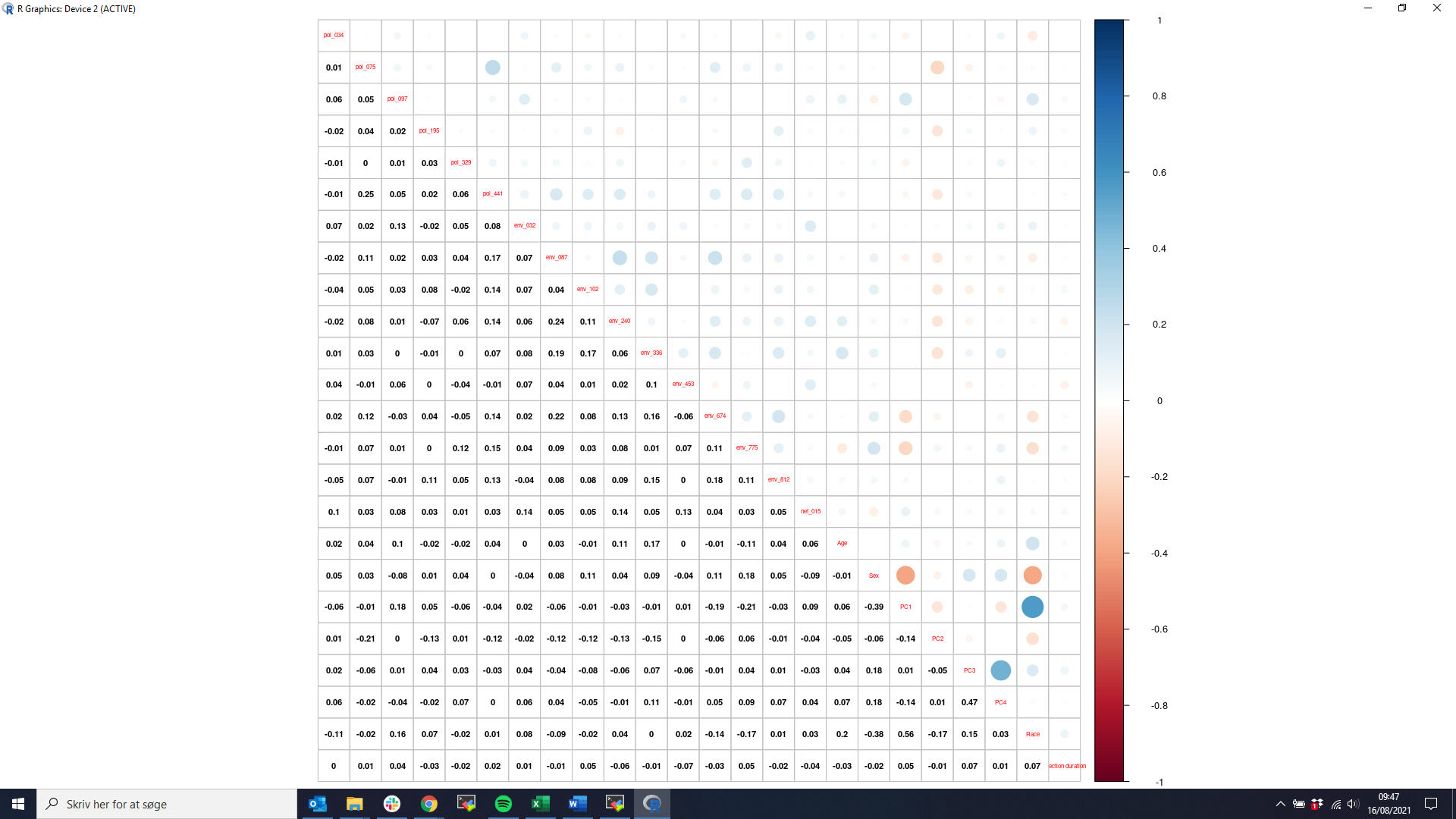


**S5 Fig.** Pairwise correlation matrix between covariates (bottom triangle).

Supplement: S5 Fig — (DOCX) [file pcbi.1010756.s013.docx]
