# Supplementary material for: Deep-sequencing of viral genomes from a large and diverse cohort of treatment-naive HIV-infected persons shows associations between intrahost genetic diversity and viral load
Source: PLoS Comput Biol. 2023 Jan 3;19(1):e1010756. doi: 10.1371/journal.pcbi.1010756 (PMC9838853; doi:10.1371/journal.pcbi.1010756)
Supplement: Attachment — Submitted filename: plos_comp_bio_rebuttal_20210304_final.docx [file pcbi.1010756.s014.docx]

Reviewer #1: Gabirelaite et al. report the results from a large cross-sectional study of a HAART-naïve HIV-1 infected cohort aimed at exploring the correlation between viral intra-host genetic diversity, analyzed by deep sequencing, and viral load, which can be considered a marker of disease progression.
Given the remarkable size and the demographic heterogeneity of the sample analyzed, this study is quite an unprecedented effort, which includes more than 2000 subjects. The scientific research question, i.e. whether the intra-host sequence diversity found in a chronically infected individual correlates with set point viral load, is very interesting and relevant both for the potential implication for predicting clinical outcome and for understanding the dynamics and the mechanism of virus evolution in vivo. Results presented show a weak, though significant, correlation with diversity, assessed by calculating Shannon Entropy. According to the study, HIV-1 env is the genetic region with the highest concentration of highly diverse position, in agreement with the high selective pressure to which Env is subjected.
Overall, I think the outcome improves our knowledge of intra-host viral evolution, contributing to the notion that intra-host diversity somehow benefits the persistence of virus replication.

*We thank the reviewer for reviewing our manuscript and the overall positive feedback.*

I wouls like to suggest a few clarifications, for the benefit of the reader:

1) What is the variability of sequencing depth of the samples that remained after excluding from analyses those with 500 or less codon positions covered by ≥500 reads? Was there an objective criterion for choosing a 500 reads threshold?

*We thank the reviewer for this question. The chosen threshold was arbitrary and based on the idea that having a minimum coverage of 500 reads at the position would allow us to call a variant of 1% frequency based on a minimum of 5 reads – enough to keep high sensitivity and confidence. The following text was added to the manuscript: “The included genomes had on average had a 5,521-fold (range 214–40,501-fold) sequencing coverage depth, and an average sequencing coverage depth of 11,638-fold (range 667–163,667-fold) for the positions included in this analysis (i.e. positions with ≥500-fold coverage depth).”*

*Also, we amended the discussion that in relation to this comment now reads: “[…] and we only included variants presented by at least 1% of covering sequencing reads which may make us disregard variation.”*

2) On a similar topic, what were the criteria for excluding positions for which ≤800 samples had mean Shannon entropy calculations? How was the 800 samples threshold decided?

*We thank the reviewer for this question. The chosen threshold was also arbitrary. The threshold was decided based on Figure 1 bottom panel that shows distinct dips in the number of samples with* *minimum 500-fold sequencing coverage depth at given positions and that there at the same time was high entropy at the positions with distinct dips in number of samples. We suspected that the distinct dips of samples with sufficient coverage may be caused by a factor, e.g. mapping of sequencing reads biased against some specific HIV-1 subtypes or subgroups of samples, that could confound our analysis. In order to better highlight and explain which positions are excluded from the analysis by this threshold we have now updated Figure 1 top panel to highlight the measured entropy of the excluded position and we have amended text to further describe the choice. We furthermore added the following text to the manuscript: “While we calculated Shannon entropy for all 2,549 HIV genomic codon positions within the targeted genomic regions (Figure 1)., 102 positions, which had sufficient sequencing coverage in ≤800 samples, were excluded from further analysis as these positions showed an aberrant number of samples with sufficient sequencing coverage and coincided with high entropy. The excluded positions were all either within Env or near the start or end of amplicons (Figure 1, Supplementary Table 2).”. Additionally, the following sentence was expanded in the limitation section: “Furthermore, success of sequencing varied across both samples and genomic positions which limited the number of samples and genomic positions that could be analyzed, and we only included variants presented by at least 1% of covering sequencing reads which may make us disregard variation.” and “Sequence reads were aligned against the HXB2 reference genome which belongs to subtype B and, therefore, some reads from other subtypes might not have been aligned at the most variable genomic regions.”*

3) given the highest concentration of positions featuring high level of diversity within Env, it would be useful to include a short comment on the location of those positions relativel to the most prominent features of Env, such as the variable loops, which could be relevant to immune escape from humoral immunity.

*We thank the reviewer for this suggestion, and we have now added the information of the prominent features for all the high entropy positions (from HIV Sequence Compendium) in Supplementary Table 2. We also performed a comparison of the differences of Shannon entropy within the variable loops of the Env and the rest of the Env. We have added the following text to the manuscript: “Mean Shannon entropy was significantly higher (Wilcoxon signed-rank test, p-value < p-value<2.2x10-16) within Env variable loop regions than in other Env positions.”.*

Reviewer #2: This paper describes the relationship between nucelotide diversity of HIV, as measured by Shannon entropy, and viral load in subjects who are treatment naiive to HIV. No major justification is given as to why this is an interesting association to study. The manuscript also discusses but does not focus on the relationship between Shannon entropy and disease progression, but perhaps underplays this positive result as it has already been described.

*We thank the reviewer for taking the time to review our work and provide helpful feedback on how it can be improved.*

Major concerns:
-The implications of finding a correlation of entropy with viral load are not clear. Perhaps if this association could explain disease progression by combination with some other data from the patients, that would be a major advance. Alternatively, the authors might focus on the uniqueness of this cohort, and their lack of treatment. Are certain loci more or less likely to be diverse than when compared to published data sets from treated individuals?-

*We thank the reviewer for this suggestion. We emphasize in the manuscript that the aim of our work is to better understand the relationship between VL (a disease progression marker) and viral intrahost diversity:*

*Specifically, our introduction reads: “we aimed to identify how Shannon entropy at AA positions as a measure of intrahost diversity varies across the HIV genome, and how it relates to the VL. We further aimed to identify the AA positions in which Shannon entropy was most-associated with the VL.”*

*Further addition of disease progression markers are indeed interesting; nonetheless, out of the scope for this manuscript as it is stated in the discussion: “more work is needed to define how intrahost genome diversity at an early point in time could be a predictor of later changes in CD4+ cell count, VL or other markers.”*

*Also, as our current study and data only include treatment-naïve persons, we cannot compare intrahost viral diversity to persons that are treated with ART, and we are not aware of any publicly available ART experienced HIV genome cohorts of comparable quality and size which could be used for comparison.*

-The authors show that diversity 'explains' VL, but the directionality of this association is not clear-- could higher VL enable more diversity?

*We thank the reviewer for this comment. While we perform an association analysis and VL is the chosen measured variable (y) we do not claim the directionality of the relationship and agree with the reviewer that the directionality could be either way and that we have no way of testing it. Therefore, we have added the following text to the manuscript: “Additionally, the directionality of the relationship between VL and intrahost diversity cannot be determined from our study”.*

*Also, we have modified two sentences to make it more clear that it is with respect to the model or the association analysis that entropy explains viral load.*

*Abstract sentence now reads “We identified 16 significant associations between VL (p-value<2.0x10-5) and Shannon entropy at AA positions which* ***in our association analysis*** *explained 13% of the variance in VL.”*

*Sentence in discussion now reads: “Finally, our results indicate that the sample’s mean Shannon entropy, especially in infections of longer duration, is informative when explaining VL variation among patients since together with other covariates* ***in our model*** *explains >10% of the overall variance in VL.”*

*Other sentences in the manuscript already included this modification, e.g.:*

*“In total,* ***the final regression model*** *(i.e., sample’s mean Shannon entropy together with all the covariates) explained 10.3% of the variance in VL.”*

*and*

*“****The full model*** *explained 21.9% of the variance in the VL.”*

-A separate test set is needed to provide confidence in model predictions (association of single AA positions and predictive ability of entropy on VL).

*We thank the reviewer for this suggestion. We agree that it would be useful to test the confidence of our model predictions and we would appreciate if the reviewer could suggest such a test set as we are not aware of any suitable public dataset. The other option to test the model would be to split our cohort into the training and testing sets but we already chose to not do it for our cohort to maintain higher statistical power. The following text was added to the discussion: “However,* *the association of VL with Shannon entropy in the 16 identified HIV amino acid positions should be further tested in other studies”*

-It would be nice to see graphs of VL vs entropy for at least of few of the associated positions. Are a few extreme points driving these associations?

*We thank the reviewer for this suggestion. We have now added the visualization (Supplementary Figure 4) of the 16 most significantly associated positions to show that the VL and entropy association is driven by the overall trend and not just a few extreme samples. We have added the following text to the manuscript: “… and in all cases the association was driven by a general trend and not a few outliers (Supplementary Figure 4)”.*

-Is VL associated with entropy at the gene level?

*We thank the reviewer for this question. We have now added this analysis to our work and added the following text to the results section: “Shannon entropy averaged per gene for each sample did not associate significantly with VL (p-value=0.31 for Gag, p-value=0.24 for Pol, p-value=0.48 for Env, p-value=0.48 for Vpu, p-value=0.33 for Rev, p-value=0.34 for Tat, p-value=0.21 for Nef).” and to the discussion: “Furthermore, our analysis of Shannon entropy averaged per gene for each sample showed that that the relationship between Shannon entropy and VL is not driven by intrahost diversity in one particular gene.”*
