# Supplementary material for: Deep-sequencing of viral genomes from a large and diverse cohort of treatment-naive HIV-infected persons shows associations between intrahost genetic diversity and viral load
Source: PLoS Comput Biol. 2023 Jan 3;19(1):e1010756. doi: 10.1371/journal.pcbi.1010756 (PMC9838853; doi:10.1371/journal.pcbi.1010756)
Supplement: Attachment — Submitted filename: plos_comp_bio_rebuttal2_20220520.docx [file pcbi.1010756.s015.docx]

Reviewer #1: I am satisfied with authors, answers and modifications in the revised manuscript.

*We thank the reviewer for their time and insights while reviewing our work*

Reviewer #2: I found some responses to my previous concerns to be unsatisfactory:
1) I understand that the authors do not want to change the scope of their manuscript, and that is certainly a reasonable choice. However, given the broad audience of this journal, it would be helpful to better explain to the reader why a relationship between VL and diversity are interesting in the abstract. It seems to me from the discussion that the authors do not believe that diversity might drive VL. Might the identified relationship allow a future researcher to model other predictors of VL by using diversity as a proxy? Is there some other utility? If not, the authors should emphasize in the abstract what they mention in the introduction – previous studies have found conflicting relationships between VL and intrahost diversity. [Minor concern]

*We thank the reviewer for these suggestions. We have now added the following text to the abstract: “Viral load is a key progression marker and understanding its relationship to viral intrahost genetic diversity could help design future strategies for HIV monitoring and treatment.”*

*While we agree that the question whether diversity drives the increase of VL or the other way around is highly relevant, our study design cannot answer it. Therefore, we mention both possibilities in the discussion section.*

*Furthermore, we mention in the abstract the conflicting results from previous studies: ”there is a disagreement whether intrahost viral genetic diversification associates positively or negatively with disease progression and progression markers.”*

2) The authors have responded to my question about specific genes being associated with VL by presenting what appears to be a fundamentally different analysis than performed for the genome as a whole (simple correlation vs multivariate regression). While a single simple correlation was done for sample mean entropy vs VL, what is reported is a correlation coefficient (and no p value), whereas only p-values (and not correlation coefficients) are reported for the gene-level analyses. [Minor concern]

*We thank the reviewer for raising this issue. The analysis performed for each gene was also a multiple regression, and we have now clarified that in the text: “Shannon entropy averaged per gene for each* ***sample in a multiple linear regression model*** *did not associate significantly with VL”. We already report p-values for each gene; no correlation coefficient is available as we performed a multiple linear regression and not Pearson’s correlation analysis.*

3) Given the centrality of the machine learning model to the data set, and the large number of positions tested, overfitting is a major possibility, particularly for the report of specific loci. I think it is appropriate and necessary for the authors to repeat their model using 800 samples as a training set and reserving 200 as a test set. The authors can compare RMSE in training and test sets and see if the same 16 positions are most correlated with VL in the test set. [Major criticism]

*We thank the reviewer for the suggestion. In this study we do not perform any machine learning analyses, we use a regression model. The reviewer’s idea to split the dataset to train and test samples could be used in the future studies. However, we think that for objective and unbiased splitting to train/test data in this study is too late since the analysis is already done and the results are obtained.*

*We have emphasized this limitation in discussion with the addition of the following sentence: “We based our analysis on the full dataset to have maximum statistical power instead of leaving a part of the dataset out for validation.”*

I also have some other concerns not mentioned in the first review:
1) The abstracts says that “genetic diversity … better disease progression marker than variation of HIV consensus sequences.” A similar claim is repeated twice in the discussion. However, I was unable to locate where this comparison emerges from, as I do not see a model presented that attempts to predict VL from consensus sequence. [Major criticism]

*We thank the reviewer for the question. This claim is based on the results from our previous work (citation 22) where we showed that HIV consensus sequences associates with VL poorly. We now have clarified in the text: “****Furthermore, in our previous work (22) by using a multiple regression model with the same covariates as in this work, we showed that VL associates poorly with HIV consensus sequences. Therefore, we conclude*** *that the HIV genome position entropy is a substantially better marker of VL than the HIV mutations identified from consensus sequences “*

2) On closer reading, I now see that the manuscript says “ In total, the final regression model (i.e., sample’s mean Shannon entropy *together with all the covariates*) explained 10.3% of the variance in VL.” How much variance in VL does a model that includes the covariates (including duration of infection!) but not mean Shannon entropy explain? In other words, how much of the performance is due to the Shannon entropy parameter? [Major criticism]

*We thank the reviewer for raising this question. The explained variance of each covariate in all of the tested models is showed in Supplementary Table 4.*

3) On page 8—“It should be noted that the samples of the excluded genomes had a significantly lower VL than the included HIV genomes”. Did coverage within the included samples also correlate with VL? This would suggest residual confounding.

*We thank the reviewer for the question. Inherently, VL and coverage are related, therefore, some level of correlation between the two measures is expected. We have now checked for the Pearson’s correlation between VL and coverage in the included samples and added this information to the text: “VL of the included samples correlated weakly with sequencing coverage (Pearson’s correlation coefficient – 0.12).”*

I also found the answer to reviewer #1s question unsatisfactory:
1) Given the per-base coverage threshold of 500 reads, it is more appropriate to exclude any samples with less than this coverage averaged across their genome.

*We thank the reviewer for the suggestion. However, since we are looking at Shannon entropy of individual HIV AA positions, the coverage threshold is required and averaged across genome threshold wouldn’t be enough since an average coverage might be high but the position’s coverage might be as low as 10x (or 0x).*

Reviewer #3: In this work, the authors study the correlation between intra-host genetic diversity during HIV-1 infections and viral load which, in turn, is a marker of disease progression. The issue is interesting from several standpoints going from a better understanding of the rules governing virus-host interactions to improving the precision of predicting clinical outcomes of the infection. The take home message of the work improves knowledge in these fields.

I have a few minor issues that the authors could address, in my opinion, to improve the manuscript.

*We thank the reviewer for their time reviewing the manuscript, helpful insights, and overall positive feedback.*

1. In the background section of the abstract they state that "viral intrahost genetic diversity remains a major obstacle to the eradication of HIV". I think that the word "eradication" is evocative of making the virus disappear from the human population. If so, I do not see a direct relationship between intra-host genetic diversity and eradication. Inter-host diversity is rather the important issue. Intra-host diversity is rather correlated to "eradication" of the virus from the patient, case for which I rather find more appropriate to use the term "cure". The same comment applies to the first line of the author summary.

*We thank the reviewer for the suggestion. We have changed the wording as suggested.*

2. Page 9, line 6 from bottom. How the conclusion that Shannon entropy accounts for 4.14% of the VL variance was reached is not straightforward (at least for me). For the sake of clarity, it should be indicated how this value (and, similarly, those indicated at lines 3 and 4 of page 10) was deduced.

*We thank the reviewer for raising this point. Explained variance calculations are described in the methods section: “The explained variance was analyzed by comparing the variable's sum of squares with the total sum of squares from analysis of variance (ANOVA) analysis.” We have also added a clarification of the used method to the results: “Sample’s mean Shannon entropy alone****, as deduced by ANOVA analysis****, explained 4.14% of the observed VL variance.” and “Sample’s mean Shannon entropy,* ***as deduced by ANOVA analysis****, explained 3.91%, 3.56%, and 10.70% of the variance in VL for infection duration of <6 months, 6–24 months and >24 months, respectively.”*

3. Also, at line 4 from the top of page 10, it is referred the effect of the interaction between Shannon entropy and duration of infection. Where is this parameter measured?

*We thank the reviewer for this question. This parameter is measured in regression model. Simply, it’s using multiplication between two parameters instead of addition, and if there is an interaction between the parameters, the multiplied parameter variable will be significant.*

4. Last sentence of the Results section. It is an interesting observation. Wouldn't it be worth to make a short comment on this in the discussion section?

*We thank the reviewer for the suggestion. We discuss this matter on page 13: “We hypothesize that these processes correlate with the acquisition of other adaptive mutations, and, therefore, higher VL (6). On the other hand, higher Shannon entropy in the associated positions could provide an advantage to the virus when avoiding the immune response, so the higher VL would be a direct outcome of the increased entropy as some of the associated positions are a part of best-defined CTL epitopes. (23,27,28). While both explanations are plausible, we did not find support that increased Shannon entropy at these positions could give rise to a direct increase in VL.”.*

5. Lines 5 to 4 from bottom of page 12. It could be interesting to add a comment for the fact that the study by Bello and coll. concerned long-term non-progressors while the present one did not (even if in that case the directionality of the correlation could be determined while in this work it could not)

*We thank the reviewer for the suggestion. We have now added to the discussion: “Our findings are in line with a smaller study by Bello et al. 2007 which found a significant positive correlation between viral genetic changes and VL in long-term nonprogressors (LTNP) (34****) even though our study was not limited to LTNP.****” and “*Additionally, while the directionality of the relationship between VL and intrahost diversity cannot be determined from our study*, the work by Bello et al. 2007 showed that increase in intrahost diversity directly leads to the increase of VL (34)”.*
