# Supplementary material for: Deep-sequencing of viral genomes from a large and diverse cohort of treatment-naive HIV-infected persons shows associations between intrahost genetic diversity and viral load
Source: PLoS Comput Biol. 2023 Jan 3;19(1):e1010756. doi: 10.1371/journal.pcbi.1010756 (PMC9838853; doi:10.1371/journal.pcbi.1010756)
Supplement: Attachment — Submitted filename: manuscript_plos_comp_bio_rebuttal3_20220825.docx [file pcbi.1010756.s016.docx]

Reviewer #2: The revised manuscript reports an association between viral load and entropy in cross-sectional HIV samples. Previous papers have found conflicting results between viral load and entropy, and this paper attempts to resolve this issue.

*We thank the reviewer for their time and effort reviewing this manuscript and for all the suggestions for the work.*

However, I have not been convinced that coverage and other artifacts have been appropriately accounted for. The authors’ apparent unwillingness to rerun the analysis with a held-out test set, or with any change of parameters (e.g. coverage threshold for sample inclusion), does not invoke confidence. If the authors are not willing to demonstrate rigor in this analysis, the impact of this finding seems low in the context of previous studies showing conflicting results.

*We appreciate the reviewer’s comment. We have added both requested analyses (rerun with a held-out test set and changing parameters) and in both cases our results and conclusions are robust.*

*First, we have added sequencing depth as a covariate in the regression models (see new Models 8 and 9 in Supplementary Table 5). The new addition to the analysis is described in the manuscript to show the effect of average sequencing depth: “Contrarily, a univariate model of log10 VL association with the average sequencing depth explained 0.68% of the variance in VL (Supplementary Table 5).” ; “If we added the average sequencing depth as a covariate to the multiple linear regression model together with other covariates, the model explained 10.7% rather than 10.3% of the variance (Supplementary Table 5).”; “We also tested how robust our results were to changes in the sequencing depth required to include positions (and thus samples) and to the addition of the average sequencing depth as a covariate to the model. Results were robust to both tests, and, while VL of the included samples correlated weakly with sequencing depth, we found average sequencing depth alone to explain little of the variance in VL.”*

*Second, we have performed a 5-fold cross validation of the regression models using both mean sample’s Shannon entropy and the position’s Shannon entropy. The following text was added to the manuscript: “To confirm that our findings are not affected by overfitting, we performed a 5-fold cross validation analysis. The average RMSE of the training and testing data set was 0.574 (0.571–0.577) and 0.578 (0.565–0.587), respectively. The explained variance was 9.3% for the test dataset and 10.4% for the train dataset.”; “Average RMSE values from train and test data in the 5-fold cross validation analysis were similar (Supplementary Table 8).”; “Finally, we obtained similar results in our analysis when we applied 5-fold cross validation, suggesting that the model was not overfitted.”*

*Third, we added the reanalysis of the data using 300x and 1000x sequencing depth thresholds (in addition to the 500x threshold of the existing analysis) to confirm that the main findings of our study stand even when using looser/stricter thresholds. The following text was added to the manuscript: “If we performed the analysis using a more stringent 1,000-fold requirement for sequencing depth of included positions (instead of the 500-fold requirement in main analysis), we identified the same positions to show high entropy; except if the positions were excluded from the analysis due to the increased requirement for sequencing depth (Supplementary Table 4). If we used a less stringent 300-fold requirement for sequencing depth, we also identified the same positions to show high entropy; yet we then also identified additional 22 high entropy positions that did not have enough sequencing depths to be included in the more stringent analyses (Supplementary Table 4).” ; “The explained variance using a 300-fold and 1000-fold sequencing depth thresholds, respectively, was comparable to the main analysis (Supplementary Table 4).”*

In addition, the abstract concludes that entropy “could be used as a better disease progression marker than HIV consensus sequence variants, especially in infections of longer duration” by pointing to a previous publication. Unless the analysis is performed on the same exact sample set (see below for one possible suggestion), this comparison should not be included prominently in the abstract.

*We thank the reviewer for the suggestion. We have now performed the analysis on the same exact sample set with the same covariates as requested and the conclusions stand the same – consensus sequences are a poor predictor of viral load. In particular, the regression model with the same data set and the same covariates resulted in 13 significant associations. All of these AA together with all the covariates in a joint model explained 6.2% of VL variance which is substantially lower than the explained VL variance of the intrahost diversity (i.e., Shannon entropy). Individually these AA variants explained 0.01-0.65% of variance in VL. Furthermore, if sequencing coverage had a strong effect of VL, the explained variance by consensus sequences should also be expected to be higher (at least comparable to Shannon entropy’s explained variance) which is clearly not the case. We added to the results section: “The regression model with the consensus AA variant calls using the same covariates showed that individual AA variants explain 0.01−0.65% of the variance in VL and the full model with the covariates explained 6.2% of the variance in VL (Supplementary Table 7).”*

Specific points:
-The authors have demonstrated that coverage correlates with VL in the data set. Yet, to my knowledge, no attempt has been made to demonstrate that coverage doesn’t impact entropy and/or correlations of entropy and VL. One way to show this would be to repeat the ANOVA analysis or linear regression in Supplementary Table 4 with coverage as a variable (and also include a model that just includes coverage for comparison with the model that just includes entropy). Another would be to remove samples with low coverage (e.g. < 500 or < 750 fold coverage) and repeat the analysis; as it stands, samples with average coverage below the per-position threshold for calculation of entropy are included, which is not best practice.

*We would like to highlight that the reviewer’s point is partly irrelevant since it is already stated in the manuscript that* *samples (<800x) and positions (<500x) with low sequencing depth are excluded from all analyses. We* ***did not*** *use any positions or samples with coverage below the thresholds for entropy calculations. The first paragraph of the results section contains more details on the thresholds used: “After read alignment to the HXB2 reference genome, 1,135 genomes had 500 or less (out of 2,549) codon positions covered by ≥500 reads. These genomes were removed from further analysis leaving 2,650 HIV genomes in the study (Supplementary Table 1). It should be noted that the samples of the excluded genomes had a significantly lower VL than the included HIV genomes (Wilcoxon signed-rank test, p-value<2.2x10-16).* ***The included genomes had on average had a 5,521-fold (range 214–40,501-fold) sequencing coverage depth, and an average sequencing coverage depth of 11,638-fold (range 667–163,667-fold) for the positions included in this analysis (i.e. positions with ≥500-fold coverage depth).****”*

*Furthermore, we performed the linear regression analysis as requested by the reviewer. We have now added to the analyses in Supplementary table 5: VL ~ average coverage and VL ~ entropy + age + sex + race + PC1-4 + infection duration + average sequencing depth. The first model explained 0.68% of variance, the second model explained 10.6% of variance which is comparable to our original model (10.3%). However, we decided not to include it in the final model since (1) the explained variance by the coverage variable alone is extremely small and (2) by including a coverage variable to the final model we would reduce the statistical power. We have also added to the manuscript text as listed above.*

-If the authors want to include a comparison of entropy vs consensus sequence prediction of viral load, an analysis of just the PCs needs to be included in Supplementary Table 4. Reference to a different but related analysis is not a sufficient replacement.

*As mentioned above, we have applied the same linear regression model (VL ~ consensus sequence + age + sex + race + PC1-4 + infection duration) to the consensus sequences to our data set and our conclusions stand the same. The following text was added to the manuscript as listed above.*

-Page 11: It is not exactly clear what is meant by “the joint model including all 16 positions”. Does this also include PCs? One possible explanation for some of the variants being unpredictive in this context is that some positions are more likely to have diversity due to alignment errors, which are more likely to occur on particular genomic backgrounds.

*Yes, the joint model includes all 16 variants and the covariates as described in supplementary table 5. The diversity due to alignment errors is irrelevant here since all the 16 positions were significant/predictive in the models with only one AA.*

Writing issues and minor suggestions:

-Page 9: What is the nature of positions in the env gene that do not have high coverage in a lot of samples? Are these impact by indels or similar?

*Highly variable regions of the env gene make the alignment to these areas hard. We added the following text to the manuscript: “102 positions, which had sufficient sequencing depth in ≤800 samples, were excluded from further analysis as these positions showed an aberrant number of samples with sufficient sequencing depth* ***(may be a result of unsuccessful mapping of reads to highly variable genomic regions)*** *and coincided with high entropy.”*

-Page 9: Why is the % of positions in each gene an important number to discuss in the results?

*We chose to remove the % for clarity now.*

-Page 10: In the results, PCs come out of seemingly nowhere. While this is mentioned in the results, the authors should walk the reader through what these are used for (genetic background of virus).

*PCA is described in more detail in the methods section. We now added that the PCs were used to “*(which are used to account for the viral genetic population structure)*”.*

-Page 10: “Bonferroni corrected p-value threshold” would be more straightforward than “our p-value threshold”

*We corrected the text as suggested.*

-Page 11: The authors should speculate on why the by-gene entropy analysis fails to associate with VL when the average of these values does associate with VL (unless I am misunderstanding what was done).

*Average sample entropy is calculated by averaging of all the positions of the sample, not by averaging gene entropies. These are two different analyses, and it is not surprising that the by-gene entropy does not associate with VL while the overall sample’s entropy associates with VL. It is likely that genes are just too short to have a significant effect of Shannon entropy association with VL.*

-Page 11: It is not exactly clear what is meant by “the joint model including all 16 positions”. Does this also include PCs?

*Yes. It includes all the AA together with all the covariates. All the details are provided in supplementary table 5.*

-Page 12: Final sentence. What evidence is being used to support this important claim?

*Supplementary Figure 4; Supplementary table 5 model 8 and 9 support this claim: “Note, that the observed results could not be explained by the fact that more variability is observed in higher sequencing depth.”*

Reviewer #3: The authors have nicely followed the suggestions made. I am happy with this revised version.

*We thank the reviewer for their time and positive feedback.*

Reviewer #4: This paper examines the relationship between intrahost viral diversity and viral load in patients enrolled in a clinical trial of early ART initiation. The study design of the parent trial means that participants tended to be either relatively early in their infection, or more likely to be relatively slow progressors, having maintained a CD4 count in the normal range (>500) without treatment. On the other hand, viral controllers are excluded from this analysis by virtue of not having sufficiently high viral load for amplification and sequencing (cut-off 1000 copies/ml).

*We thank the reviewer for their insights while reviewing our work.*

Although previous studies have suggested that the extent of intrahost viral diversity is likely to vary over the course of HIV infection (for example, being lower in late disease when host immune responses are less effective), this is a cross-sectional rather than a longitudinal study, with only a single sample being studied in each patient. I think there are some concerns about trying to relate this to viral load in patients early in infection, during the very dynamic stages of primary HIV infection - in many ways I would be more comfortable if the analysis focused on patients who had already reached set-point viral load (usually some time between 6 and 24 months), although I appreciate that the authors have calculated the mean Shannon entropy separately for patients infected less than 6 months, 6-24 months and >24 months. I think it is also important to note that the amplicons do not cover the full length of the HIV genome, missing out p17 as well as vif and vpu.

*We agree that time since infection could impact the VL, therefore, in all of the models the infection duration covariate is included to correct for it. Complete exclusion of these samples in unnecessary. The fact that amplicons do not cover the full genome is emphasized in both methods and results section: we indeed do not have sequencing data on p17 (part of gag), vif and vpr genes, vpu is within the amplicons and sequenced. Finally, the incomplete genome sequencing is also marked in the figures.*

I am not convinced that the debate about the relationship between viral diversity and viral load in HIV infection is as intense as the authors suggest. If this is a key argument about the novelty of this paper, then I think the authors should have made more effort to review the literature about HIV diversity and viral load/clinical outcome, focusing on the major studies on this topic. A number of key papers, which were not cited, have come to the same conclusions as the authors, for example: Esbjornsson et al, NEJM, 2012 (which is a longitudinal study that demonstrates increasing diversity over time up to a “diversity threshold” heralding the onset of AIDS), and a very similar study to this one, by Blanquart et al in PLOS Biology 2017, in which the authors focus specifically on the early stages of HIV infection and conclude that viral diversity contributes around one third of set-point viral load variability.
In contrast, the main argument to the contrary was reported by Wolinsky et al (Science 1996) in a select group of patients with very rapid disease progression, who were assumed to have mounted very limited immune responses to the virus.

*We would like to emphasize that the whole work, including the argument in question, is about the intrahost viral diversity, i.e. identification of low frequency variants and association of their abundance with disease progression through VL rather than consensus sequence diversity. The suggested papers are indeed of high relevance for viral diversity and it’s association with VL; however, it is not related with the key topic of our work.*

I don't think anyone in the HIV field will be surprised to hear that the greatest diversity is found in the env gene, so the main novelty in the paper is the identification of amino acids where variation correlates significantly with viral load: several of these are found in the vicinity of T-cell epitopes but the relationship between env amino acids and antibody-binding epitopes has not been considered - I think this would add considerably to the paper (see, for example, Andrews et al, Scientific Reports, 2018).

**Have the authors made all data and (if applicable) computational code underlying the findings in their manuscript fully available?**
The [PLOS Data policy](https://journals.plos.org/ploscompbiol/s/materials-and-software-sharing) requires authors to make all data and code underlying the findings described in their manuscript fully available without restriction, with rare exception (please refer to the Data Availability Statement in the manuscript PDF file). The data and code should be provided as part of the manuscript or its supporting information, or deposited to a public repository. For example, in addition to summary statistics, the data points behind means, medians and variance measures should be available. If there are restrictions on publicly sharing data or code —e.g. participant privacy or use of data from a third party—those must be specified.

Reviewer #2: **No:** A promise is made to upload these items, but this has not yet been delivered on.

Reviewer #3: None

Reviewer #4: Yes

*All the relevant data and code is made available on Dryad repository: https://datadryad.org/stash/dataset/doi:10.5061/dryad.6djh9w13s. Furthermore, we have now further updated the repository with the raw codon calls from VirVarSeq to the repository.*
