# Supplementary material for: Deep-sequencing of viral genomes from a large and diverse cohort of treatment-naive HIV-infected persons shows associations between intrahost genetic diversity and viral load
Source: PLoS Comput Biol. 2023 Jan 3;19(1):e1010756. doi: 10.1371/journal.pcbi.1010756 (PMC9838853; doi:10.1371/journal.pcbi.1010756)
Supplement: Attachment — Submitted filename: manuscript_plos_comp_bio_rebuttal4_20221028.docx [file pcbi.1010756.s017.docx]

Reviewer #2: The manuscript has been much improved in terms of rigor by the addition of new analyses. While cross-validation is not the same as an independent test set, the direct analyses of the effects of coverage and sequence validation has satisfied my concerns.

I do not want to unnecessarily burden the authors, but one thing the authors and editor might consider together is improving the readability of the manuscript by including a figure in the main text that summarizes the models tried and the relative predictive power of these models. Having to go to the supplement to understand the evidence for key claims (in particular the relative predictive power of sequence/PC and entropy, mentioned in abstract) is not ideal.

*We thank the reviewer for their time throughout the whole review process and for their contributions towards improving the quality of our work. We feel that peer-reviewers’ comments and suggestions helped markedly.*

*We have now added a table which summarizes the main findings as suggested by reviewer (Table 2).*

Reviewer #4: The authors have addressed only one of my comments, about the influence of time since infection on the analysis, and have either ignored or failed to address the other comments. I think that the question of the novelty of their findings remains to be answered.

*We thank the reviewer for their time reviewing the manuscript and raising these concerns.*

*As pointed out by the reviewer, the debate on whether viral diversity has a positive or negative relationship with disease progression may not anymore be as intense as highlighted by earlier papers (e.g., our reference 3: Lee et al.,* PLOS Computational Biology*, 2008,* [*https://doi.org/10.1371/journal.pcbi.1000240*](https://doi.org/10.1371/journal.pcbi.1000240)*). Accordingly, we have removed our highlight of this debate from the manuscript, and we have added the following texts to better put the results into context of existing literature and to clarify the novelty of our study and findings:*

*Abstract:*

*“the association between intrahost diversity and disease progression markers has not been investigated in large and diverse cohorts for which the majority of the genome has been deep-sequenced”*

*“Finally, equivalent analysis based on variation in HIV consensus sequences explained only 2% of VL variance.*

*Author summary:*

*“there is a lack of large and diverse cohort studies based on near-full and deep-sequenced HIV genomes”*

*“whereas equivalent analysis based on variation in HIV consensus sequences explained only 2% of VL variance.”*

*Introduction:*

*“To date, the relationship between intrahost genetic diversity and disease progression markers, e.g. viral load (VL), have been investigated in relatively highly selected and small cohorts (up to 187 individuals in Hightower et al., 2012 (10)) and have typically been based on techniques that allows for only low depth sampling for variation in a minor part of the HIV genome (10,12,14-22). Investigations based on near-full and deep genome sequencing of HIV genomes from large and diverse cohorts are required to further elucidate the relationship between intrahost genetic diversity and disease progression and to generalize previous findings from smaller and more homogenous cohorts.*

*“Participants from across 35 countries in the Strategic Timing of AntiRetroviral Treatment (START) clinical trial represent a demographically diverse cohort of treatment naïve HIV-positive persons infected with a range of different HIV subtypes and for which near-full viral genomes have been deep sequenced (23-26).”*

*“and finally, we compared whether interhost or intrahost HIV genetic variation better explained variance in VL.”*

*Discussion:*

*“While we and others have associated variation in consensus sequences of the HIV-1 quasispecies with disease progression markers in studies with >1,000 individuals (5,25,34), equivalent analyses of intrahost viral diversity have, despite showing strong associations, been conducted on cohorts with only up to 187 individuals (10) and have typically been based on low depth sampling of genetic variation in only a minor part of the HIV genome (10,12,14-22).”*

*“Consequently, our study addresses the need for associations between intrahost diversity and disease progression markers in a large and demographically diverse cohort with near-full deep-sequenced HIV genomes. Moreover, we compare the strength of the association between VL and intrahost diversity with VL and consensus HIV AA variants (Table 2).”*

*Finally, we have changed the title from “Deep-sequencing of viral genomes from treatment-naive HIV-infected persons shows positive association between intrahost genetic diversity and viral load” to “Deep-sequencing of viral genomes from a large and diverse cohort of treatment-naive HIV-infected persons shows associations between intrahost genetic diversity and viral load”. Again, this is to clarify that our study should not be seen as a response to a debate on whether viral diversity is positively or negatively associated with disease progression, but rather the improved title better tells that our study is using a large and diverse dataset to investigate multiple aspects and detail-levels of the association between viral diversity and a disease progression marker.*

*Note, we have with the amendments added the following references to the manuscript:*

*Silver N, Paynter M, McAllister G, Atchley M, Sayir C, Short J, et al. Characterization of minority HIV-1 drug resistant variants in the United Kingdom following the verification of a deep sequencing-based HIV-1 genotyping and tropism assay. AIDS Res Ther. 2018 Nov 8;15(1):18.*

*Wolinsky SM, Korber BT, Neumann AU, Daniels M, Kunstman KJ, Whetsell AJ, et al. Adaptive evolution of human immunodeficiency virus-type 1 during the natural course of infection. Science. 1996 Apr 26;272(5261):537–42.*

*Esbjörnsson J, Månsson F, Kvist A, Isberg P-E, Nowroozalizadeh S, Biague AJ, et al. Inhibition of HIV-1 disease progression by contemporaneous HIV-2 infection. N Engl J Med. 2012 Jul 19;367(3):224–32.*

*Shankarappa R, Margolick JB, Gange SJ, Rodrigo AG, Upchurch D, Farzadegan H, et al. Consistent viral evolutionary changes associated with the progression of human immunodeficiency virus type 1 infection. J Virol. 1999 Dec;73(12):10489–502.*

*Nowak MA, Anderson RM, McLean AR, Wolfs TF, Goudsmit J, May RM. Antigenic diversity thresholds and the development of AIDS. Science. 1991 Nov 15;254(5034):963–9.*

*De Troyer A, Kirkwood PA, Wilson TA. Respiratory action of the intercostal muscles. Physiol Rev. 2005 Apr;85(2):717–56.*

*Markham RB, Wang WC, Weisstein AE, Wang Z, Munoz A, Templeton A, et al. Patterns of HIV-1 evolution in individuals with differing rates of CD4 T cell decline. Proc Natl Acad Sci USA. 1998 Oct 13;95(21):12568–73.*
