# Supplementary material for: Deep-sequencing of viral genomes from a large and diverse cohort of treatment-naive HIV-infected persons shows associations between intrahost genetic diversity and viral load
Source: PLoS Comput Biol. 2023 Jan 3;19(1):e1010756. doi: 10.1371/journal.pcbi.1010756 (PMC9838853; doi:10.1371/journal.pcbi.1010756)
Supplement: Attachment — Submitted filename: manuscript_plos_comp_bio_rebuttal5_20221122.docx [file pcbi.1010756.s018.docx]

*We thank the reviewers for all their time and efforts to help improve the manuscript.*

Reviewer #2: I appreciate that the authors attempted to meet my request for a Table 2 for a direct comparison of models. However, this table is confusing and not helpful in its current form-- as it is hard to compare prose across the various models. Information on entropy should be removed (this doesn't need to summarize the entire paper!) and versions of the same exact model should be compared in a simple table, where each row corresponds to the data included in that model and the value presented in each cell shows the total performance of the model (e.g. % of VL variance explained). Values of table should only include numbers, and explanations of what these numbers mean should be included in the headers of the column(s) (indicating that the equivalent metrics are being compared across models).

Alternatively, the authors could remove this table.

*We have now simplified Table 2 as requested by the reviewer to only include the model and its explained variance.*

Reviewer #4: I am glad that the authors have now addressed my comments.

*We thank the reviewer for their comments and positive feedback.*

*Additionally, we corrected the supplementary table order, corrected one reference (21), added a missing cross-reference to Supplementary Table 6 and added additional information to Supplementary Table 8 to make it show same detail-level as Supplementary Table 6.*
